# Automated measurement of leakage on wide-field angiography in the assessment of retinal vasculitis

**DOI:** 10.1186/s12348-019-0193-8

**Published:** 2020-02-03

**Authors:** Arthi G. Venkat, Sumit Sharma

**Affiliations:** Cleveland Clinic, Cole Eye Institute, 9500 Euclid Avenue, Desk i32, Cleveland, OH 44195 USA

**Keywords:** Automated analysis, Ultra-wide-field fluorescein angiography, Posterior uveitis, Retinal vasculitis

## Abstract

Automated analysis of leakage on fluorescein angiography is a measurable and clinically applicable endpoint that can be used to follow patients with posterior uveitis. A number of studies have analyzed the use of automated analysis of leakage on fluorescein angiography and are reviewed in this article.

## Background

Posterior uveitis is diagnosed and monitored by a combination of clinical findings and multimodal imaging. However, current established grading systems for disease monitoring are inconsistent and unable to provide objective, numerical endpoints. Clinical findings in posterior uveitis such as vitreous cells and haze may be helpful but are difficult and arduous to grade consistently in an objective manner despite established clinical grading systems.

Multimodal imaging augments the study of uveitis by allowing us to visualize endpoints that are not clinically apparent. Retinal vascular leakage is an endpoint that can only be visualized using fluorescein angiography. Multiple studies have demonstrated the concordance of angiographic leakage with inflammatory activity [1, 2], and ultra-wide-field (UWF) angiography has demonstrated improved detection of peripheral vascular leakage (PVL) [1–6].

Retinal vascular leakage, although useful, is difficult to assess efficiently and with consistent accuracy in the clinical setting. Factors such as image exposure, imaging angles, and focus can lead the clinician to over- or under-estimate the amount of leakage present. The process of assessing angiographic leakage in the clinical setting can be time-consuming and cumbersome. Additionally, without the use of readily quantifiable endpoints, clinical trials in uveitis are limited in their ability to demonstrate the improvement of measurable variables. Arguably, this has limited drug development in uveitis due to failure to meet primary endpoints that are difficult to assess, such as vitreous haze [7].

The development of software that can transform retinal vascular leakage into a quantifiable endpoint is one of many advances in automated analysis. Quantification of imaging endpoints can provide ordinal variables at specific timepoints and continuous variables that can be followed over time to determine changes in both the clinical and research setting. Using software to analyze angiographic leakage as a numeric endpoint has demonstrated utility in non-inflammatory disease such as diabetic retinopathy [8]. The use of automated leakage analysis in inflammatory disease is less studied but can provide a powerful variable by which to monitor disease.

## Wide-field angiography and posterior uveitis

**Fig. 1:**
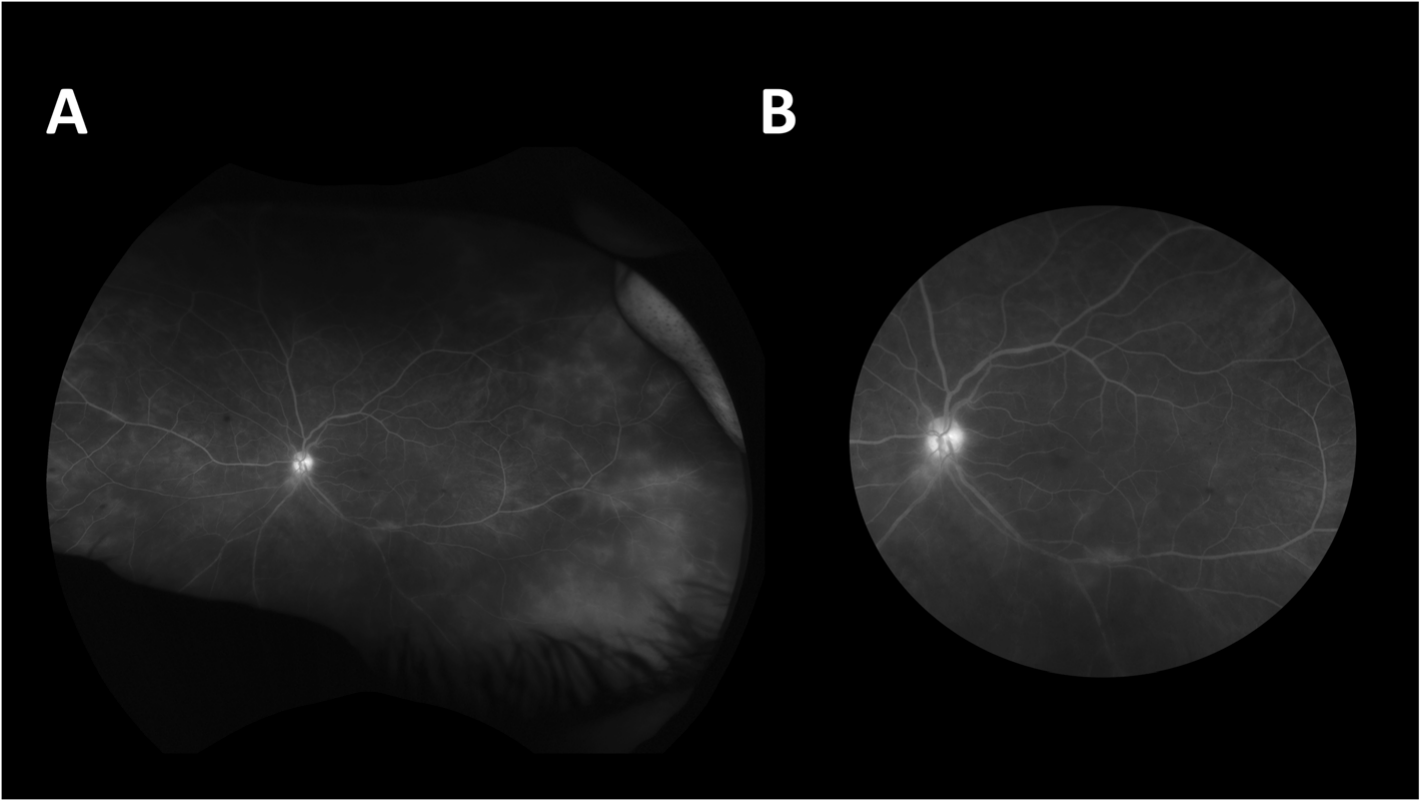
**a** Ultra-wide-field (200-degree) fluorescein angiography (FA) image. **b** Image cropped to simulated 30-degree conventional FA in a patient with posterior uveitis

In comparison with conventional angiography, ultra-wide-field angiography is able to capture a greater area of the retina. The advent of ultra-wide-field (UWF) imaging has greatly augmented the visualization of PVL, with some platforms providing a view of the retina over 200 degrees compared with the conventional degree fluorescein angiography (FA) which usually spans 30 to 60 degrees. Figure 1 demonstrates the significant difference in the amount of leakage on ultra-wide-field fluorescein angiography (UWFFA) image compared with an image cropped to a simulated conventional field in an eye with posterior uveitis.

PVL as a prognostic factor in uveitis has been studied and, although not found to impact visual acuity over one year or less [4], has demonstrated impact on therapeutic decision-making [1, 5].

Additionally, previous literature has shown PVL to correlate to clinical activity as determined by the clinician based on examination [9] and to the presence of cystoid macular edema [10]. Based on this, retinal vascular leakage is likely an important surrogate endpoint in uveitic disease activity.

Studies have demonstrated the superior ability of UWF imaging to provide clinically significant information compared with standard field FA [1–3, 5]. Pecen and colleagues [1] demonstrated in a retrospective review that ultra-wide-field fluorescein angiography (UWFFA) revealed increased leakage in comparison with simulated 50-degree FA images, and found that peripheral vascular leakage was missed in 27% of eyes without the UWFFA image. Campbell et al. conducted a prospective study that found a 32% change in management with the addition to UWFFA, compared with clinical examination and simulated conventional FA alone. In addition, this study also found disease activity in 63% of patients with UWFFA, compared with 51% based on examination and simulated conventional FA alone [3].

Scanning laser ophthalmoscope (SLO) UWFFA (i.e., Optos) has also been shown to demonstrate greater peripheral retinal vascular leakage compared with 9-field montage images, even in peripheral regions adequately imaged by both cameras [6]. The increased sensitivity of UWFFA in detecting retinal vascular leakage is postulated to be due to a combination of the coaxial lighting system and the suppression of extraneous light by filters, thus suppressing background fluorescence and allowing detection of subtle leakage.

These advantages of UWFFA make it an ideal imaging modality to detect and monitor retinal vascular leakage over time. Therefore, the use of automated leakage analysis is best done with UWFFA and has demonstrated the ability to provide a quantifiable disease endpoint.

## Quantitative leakage analysis

The quantification of retinal vascular leakage on UWFFA can provide a strong visual and numerical endpoint for diagnosis and disease monitoring. In order to create a validated automated quantification algorithm, previous studies have laid the groundwork for the automated identification of angiographic leakage. A study of patients with peripheral vasculitis, ischemia, and vascular leakage was performed in which areas of peripheral leakage or ischemia were manually identified, then quantified as a percentage of the total FA area [10]. However, manual segmentation and leakage identification are impractical in the clinical practice setting.

**Fig. 2:**
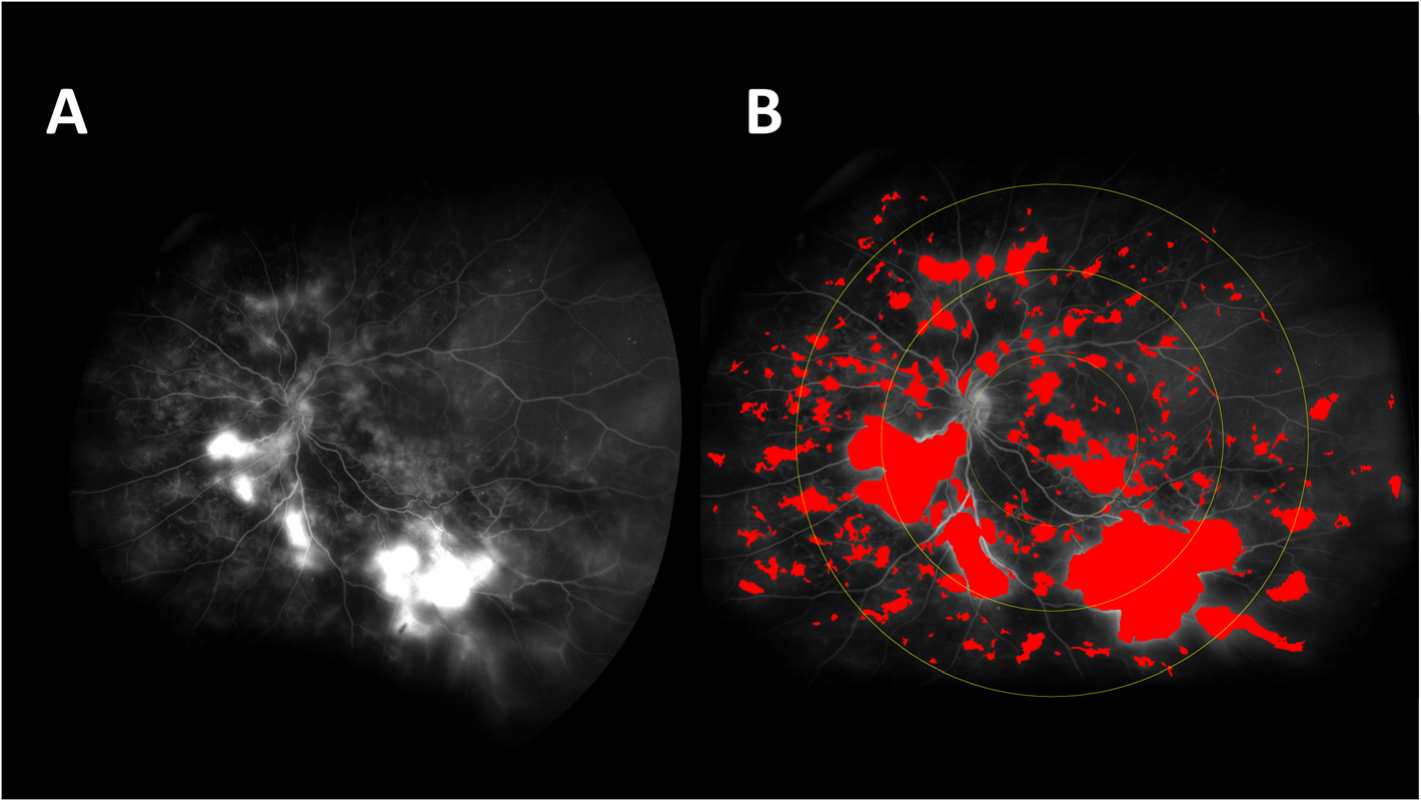
**a** Ultra-wide-field FA image of patient with proliferative diabetic retinopathy (PDR). **b** Pseudocolor mask created from regions of interest (ROIs) using automated leakage analysis software

More recently, Ehlers et al. published an automated quantification algorithm for UWFFA images in diabetic patients [8]. The algorithm was developed using expert readers performing manual quantification to provide iterative feedback. Figure 2 demonstrates the application of the algorithm to the fluorescein angiogram of a diabetic patient.

Figure 2a displays the late-phase angiogram of a patient with proliferative diabetic retinopathy. The program uses retinal vascular patterns for image registration, and later to remove vessels when regions of interest are identified. Late-phase angiography images are flattened and de-warped to eliminate distortion from the curvature of the eye, the late-phase image is compared with an early-phase image, and leakage areas are equalized in intensity to use a fixed threshold by which the algorithm may identify them as regions of interest (ROI). A pseudocolor mask is then created by the ROIs and superimposed onto the late-phase image (Fig. 2b). Based on the ROIs detected, a leakage index is calculated as a means of quantifying the leakage numerically. Fluorescence that is detected by the algorithm must have a minimum intensity equivalent to that of adjacent vasculature, which allows the distinction of true leakage from background fluorescence. The algorithm demonstrated a strong correlation between the automated protocol and manual quantification.

## Applications of automated leakage quantification in posterior uveitis

**Fig. 3:**
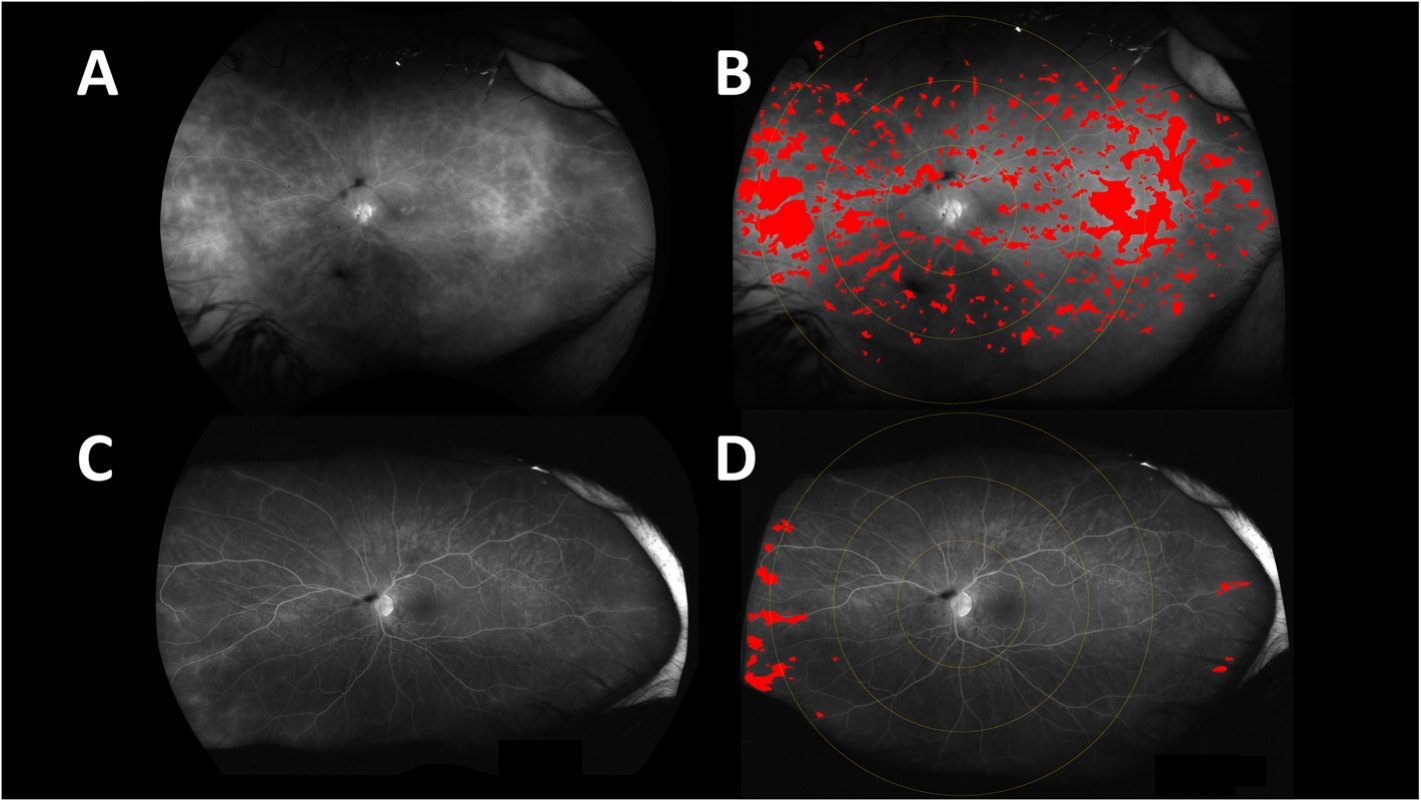
Ultra-wide-field FA image pre (**a**) and post (**b**) bilogic therapy in a patient with posterior uveitis. Corresponding authomated leakage analysis with pseudocolor masks (**c** and **d**)

Using such an algorithm, images of patients with posterior uveitis can be similarly analyzed. Figure 3 shows UWFFA images prior to and following therapy with the biologic adalimumab in a patient with non-infectious posterior uveitis. Images A and B display the untouched late UWFFA images pre and post biologic therapy, and Images C and D show the masks of pseudo-colored regions of leakage. The pre- and post-treatment images demonstrate a clear decrease in the size of areas as evidenced by a reduction in the size of the pseudocolor mask as well as a reduction in the leakage index by 13.6% over the course of 6 months of therapy [11].

## Conclusion

Posterior uveitis in many cases can be most accurately monitored using clinical endpoints not visible to the naked eye, namely angiographic leakage. Automated leakage analysis turns retinal vascular leakage into a readily measurable and clinically applicable endpoint that would greatly expedite and simplify the task of FA interpretation. Automated analysis also eliminates subjectivity bias, allowing the clinician to use this ordinal endpoint to augment clinical analysis. By combining a large amount of information gleaned using UWFFA with the expediency of automated analysis, better outcomes can be obtained for complex uveitis patients.

## Data Availability

Not applicable.

## Abbreviations

FA: Fluorescein angiography
PDR: Proliferative diabetic retinopathy
ROI: Regions of interest
SLO: Scanning laser ophthalmoscope
UWF: Ultra-wide field
UWFFA: Ultra-wide-field fluorescein angiography
